# Supplementary figures and images for: Dual gene set enrichment analysis (dualGSEA); an R function that enables more robust biological discovery and pre-clinical model alignment from transcriptomics data
Source: Sci Rep. 2024 Dec 4;14:30202. doi: 10.1038/s41598-024-80534-8 (PMC11618328; doi:10.1038/s41598-024-80534-8)

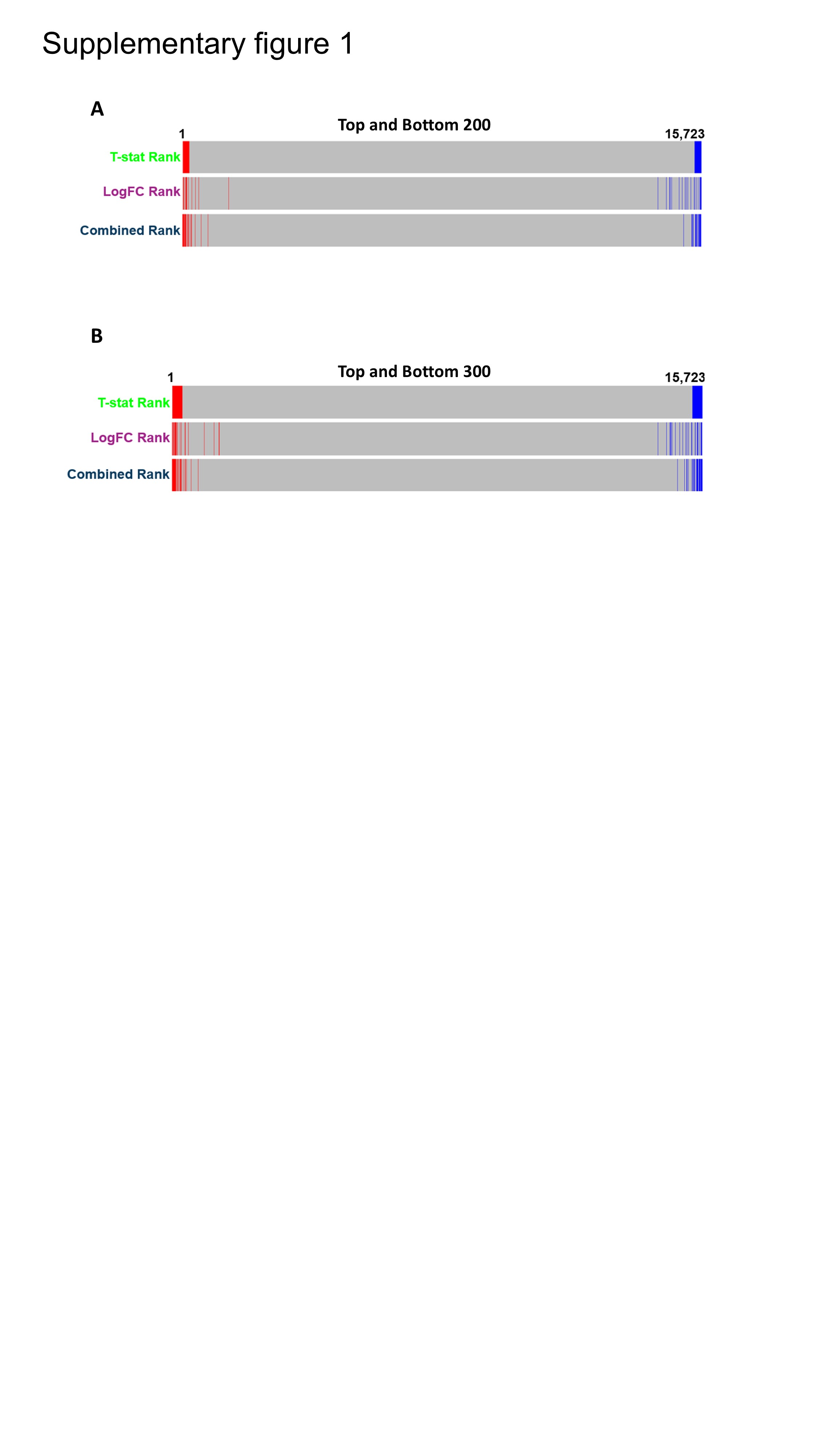

Supplement: Supplementary file 1 — Supplementary Material 1 [file 41598_2024_80534_MOESM1_ESM.jpg]

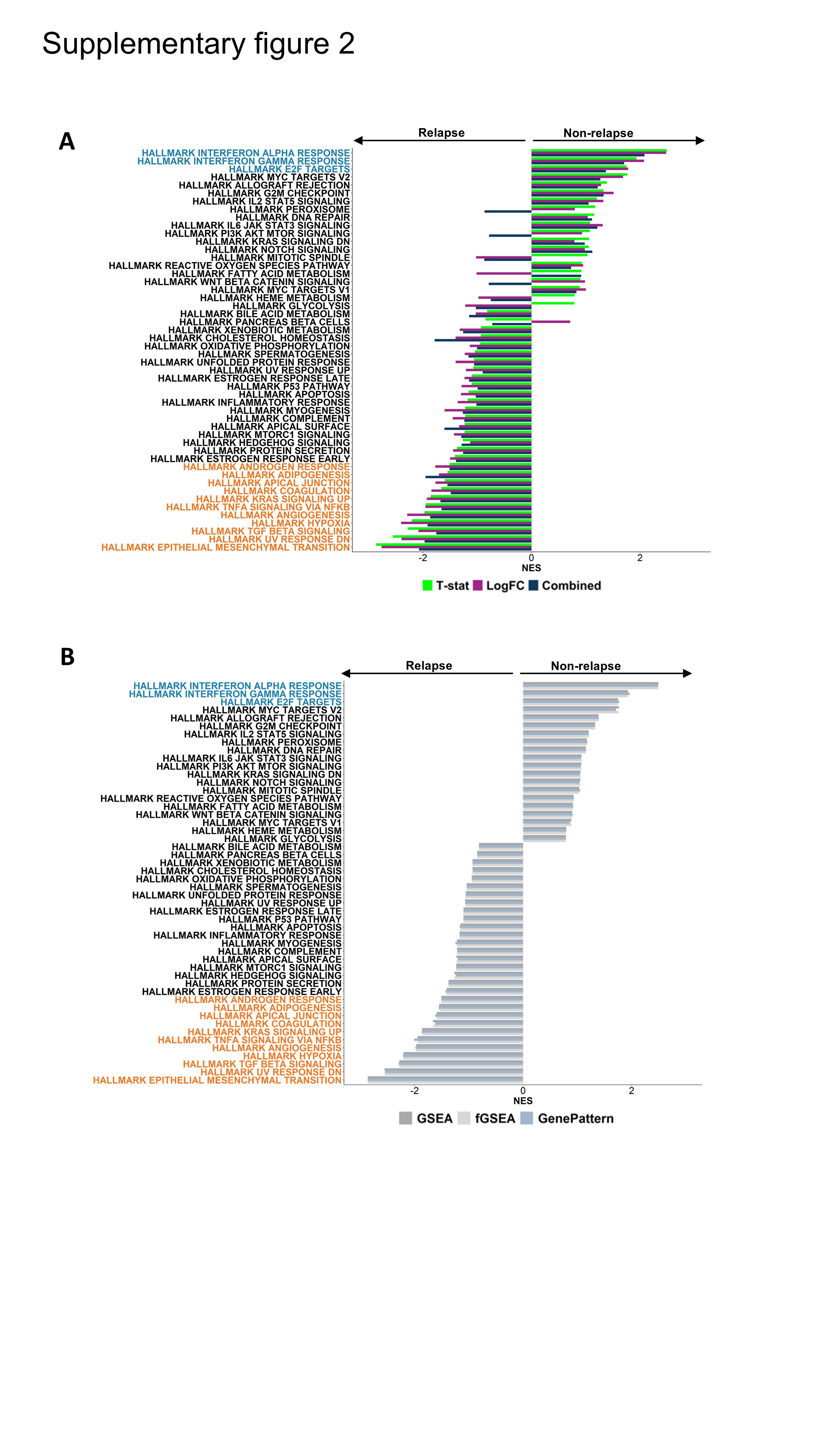

Supplement: Supplementary file 2 — Supplementary Material 2 [file 41598_2024_80534_MOESM2_ESM.jpg]

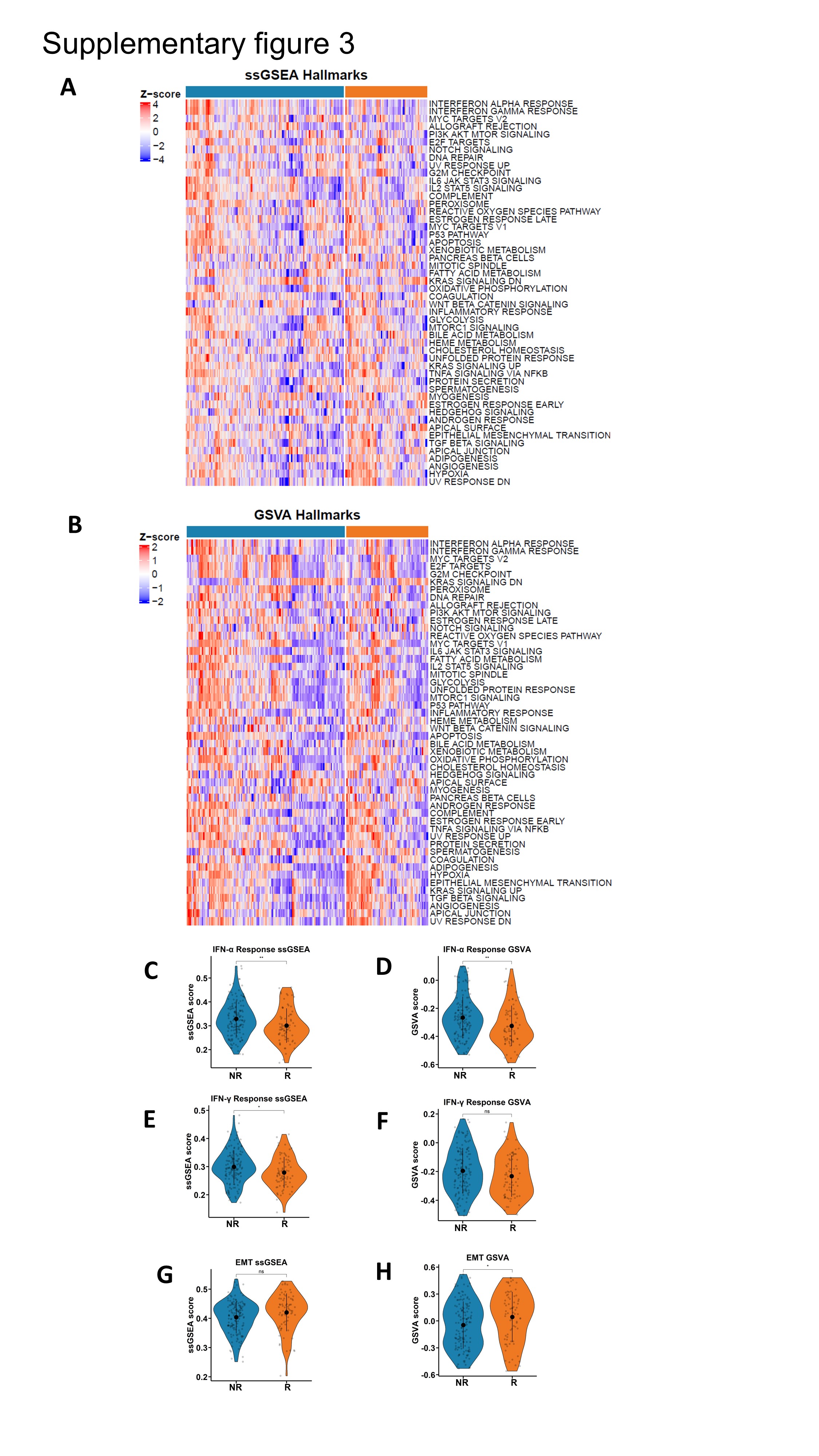

Supplement: Supplementary file 3 — Supplementary Material 3 [file 41598_2024_80534_MOESM3_ESM.jpg]

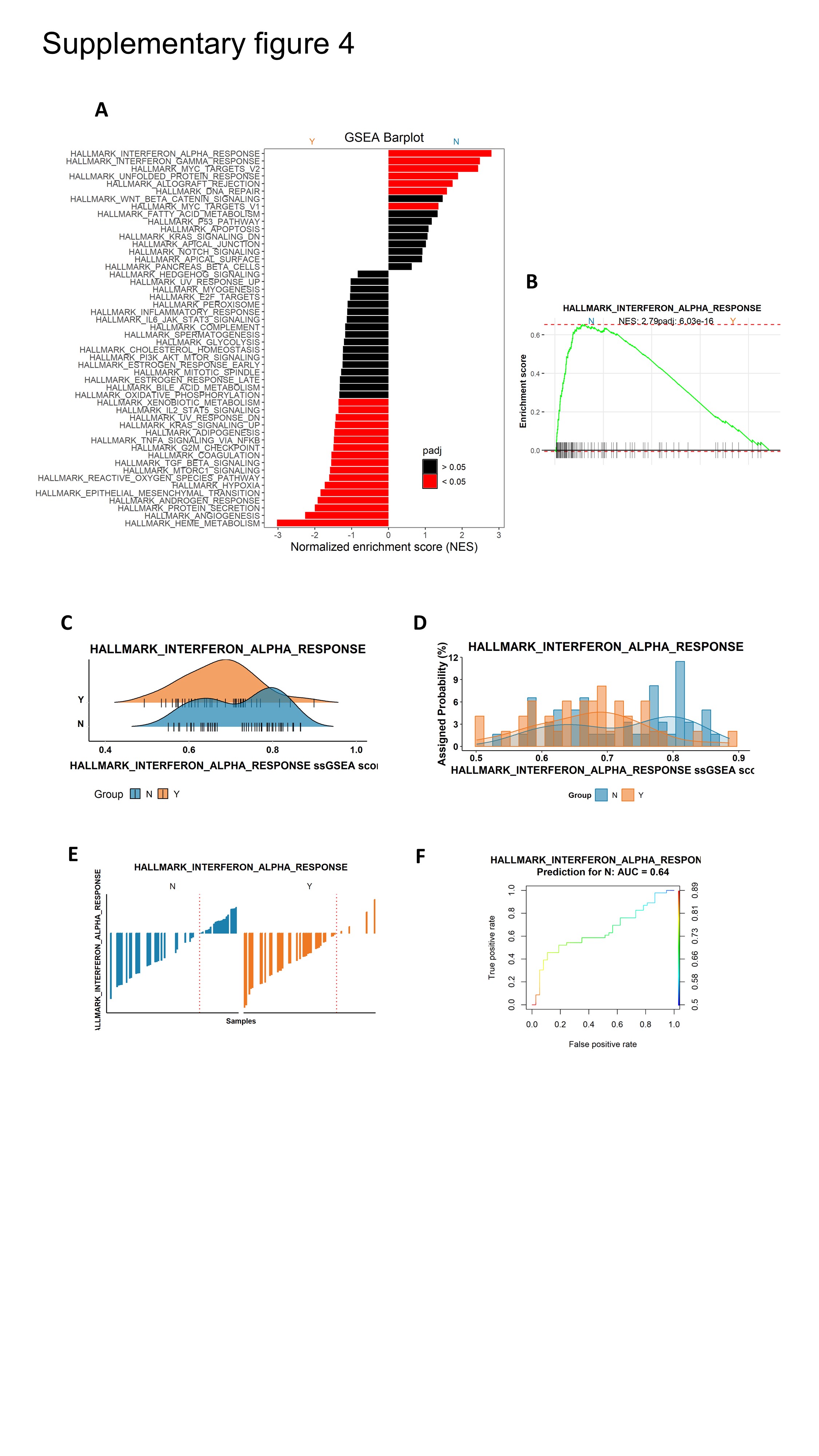

Supplement: Supplementary file 4 — Supplementary Material 4 [file 41598_2024_80534_MOESM4_ESM.jpg]
