# Supplementary material for: Dual gene set enrichment analysis (dualGSEA); an R function that enables more robust biological discovery and pre-clinical model alignment from transcriptomics data
Source: Sci Rep. 2024 Dec 4;14:30202. doi: 10.1038/s41598-024-80534-8 (PMC11618328; doi:10.1038/s41598-024-80534-8)
Supplement: Supplementary file 5 — Supplementary Material 5 [file 41598_2024_80534_MOESM5_ESM.docx]

**Supplementary Figure Legends:**

**Supplementary figure 1. Comparison of the top and bottom differentially expressed genes across *t*-stat, LogFC and combined ranking metrics. (A)** Ranked position of the top 200 differentially expressed genes and bottom 200 genes in NR when ranked by *t*-stat and the position of these genes when ranked by LogFC and combined. **(B)** Ranked position of the top 300 differentially expressed genes and bottom 300 genes in NR when ranked by *t*-stat and the position of these genes when ranked by LogFC and combined. Top genes indicated in “red” and bottom genes indicated in “blue”.

**Supplementary figure 2. Ranking metrics of differentially expressed genes for GSEA have little impact on GSEA results and GSEA methods have little variation. (A)** 50 Hallmark gene sets from clusterProfiler GSEA when genes were ranked by *t*-stat, LogFC, and combined, highlighting the significant (padj < 0.05) hallmarks that are associated with all three ranking methods. **(B)** clusterProfiler GSEA, fGSEA, and GenePattern pre-ranked GSEA 50 Hallmark gene sets ranked by *t*-stat. Labels of statistically significant gene sets are highlighted in “blue” for non-relapse and “orange” for relapse.

**Supplementary figure 3. Comparison of single sample analysis methods. (A)** ssGSEA heatmap of 50 Hallmark gene sets. **(B)** GSVA heatmap of 50 Hallmark gene sets. **(C)** Significance between NR and R ssGSEA scores for Interferon Alpha Response (** padj < 0.01) **(D)** Significance between NR and R GSVA scores for Interferon Alpha Response (** padj < 0.01). **(E)** Significance between NR and R ssGSEA scores for Interferon Gamma Response (* padj < 0.05). **(F)** No significance between NR and R GSVA scores for Interferon Gamma Response (ns). **(G)** No significance between NR and R ssGSEA scores for EMT (ns**) (H)** Significance between NR and R GSVA scores for EMT (* padj < 0.05).

**Supplementary figure 4. Application of *dualgsea* in an independent non-cancer dataset, GSE213313. (A)** Bar plot of Hallmark gene sets to compare patients with critical disease (Y) to patients with non-critical disease (N). **(B)** Enrichment plot of GSEA indicating enrichment of Hallmark Interferon Alpha Response in N patients compared to Y patients. ssGSEA scores show large overlap between Y and N patients with **(C)** density plot and **(D)** histogram. **(E)** Stratification of Hallmark Interferon Alpha Response show similar proportions of low scores for Y and N patients. **(F)** ROC indicates AUC of 0.64 when predicting for N patients using Hallmark Interferon Alpha Response scores.
